# Preconditioning of Mesenchymal Stem Cells by Sevoflurane to Improve Their Therapeutic Potential

**DOI:** 10.1371/journal.pone.0090667

**Published:** 2014-03-05

**Authors:** Xuejun Sun, Bo Fang, Xi Zhao, Guangwei Zhang, Hong Ma

**Affiliations:** 1 Department of Anesthesiology, First Affiliated Hospital, China Medical University, Shenyang, Liaoning, China; 2 Department of Cardiac Surgery, First Affiliated Hospital, China Medical University, Shenyang, Liaoning, China; The University of Adelaide, Australia

## Abstract

**Background:**

Bone marrow mesenchymal stem cells (MSCs) have been found to produce beneficial effects on ischemia-reperfusion injury. However, most of the MSCs died when transplanted into the ischemic tissue, which severely limit their therapeutic potential.

**Methods:**

Using an in vitro model of hypoxia and serum deprivation (H/SD), we investigated the hypothesis that sevoflurane preconditioning could protect MSCs against H/SD-induced apoptosis and improve their migration, proliferation, and therapeutic potential. The H/SD of MSCs and neuron-like PC12 cells were incubated in a serum-free medium and an oxygen concentration below 0.1% for 24 h. Sevoflurane preconditioning was performed through a 2-h incubation of MSCs in an airtight chamber filled with 2 vol% sevoflurane. Apoptosis of MSCs or neuron-like PC12 cells was assessed using Annexin V-FITC/propidium iodide (PI). Furthermore, the mitochondrial membrane potential was assessed using lipophilic cationic probe. The proliferation rate was evaluated through cell cycle analysis. Finally, HIF-1α, HIF-2α, VEGF and p-Akt/Akt levels were measured by western blot.

**Results:**

Sevoflurane preconditioning minimized the MSCs apoptosis and loss of mitochondrial membrane potential. Furthermore, it increased the migration and expression of HIF-1α, HIF-2α, VEGF, and p-Akt/Akt, reduced by H/SD. In addition, neuron-like PC12 cells were more resistant to H/SD-induced apoptosis when they were co-cultured with sevoflurane preconditioning MSCs.

**Conclusion:**

These findings suggest that sevoflurane preconditioning produces protective effects on survival and migration of MSCs against H/SD, as well as improving the therapeutic potential of MSCs. These beneficial effects might be mediated at least in part by upregulating HIF-1α, HIF-2α, VEGF, and p-Akt/Akt.

## Introduction

Bone marrow mesenchymal stem cells (MSCs) transplantation is an attractive therapeutic method for tissue injuries, such as myocardial infarction [1] as well as cerebral and spinal cord ischemia [2], [3]. However, this treatment is limited due to the low survival rate of MSCs after transplantation. Most of the grafted MSCs died due to apoptosis in the early stages of transplantation [4], [5]. Therefore, it was necessary to protect MSCs against apoptosis during transplantation. Various strategies have been studied to enhance the survival of the transplanted cells. Preconditioning MSCs with pharmacological agents might produce a comparable cytoprotective effect against hypoxia with hypoxic preconditioning [6]–[9].

Sevoflurane, a novel inhaled anesthetic, is widely used in clinical anesthesia. Many studies have demonstrated that sevoflurane preconditioning and postconditioning could induce an ischemic tolerance against ischemic injury at an organic or cellular level [10]–[13]. Moreover, recent studies have reported that sevoflurane preconditioning could promote the growth and proliferation of the stem cell-like human endothelial progenitors and increased the mobilization of the bone marrow mononuclear cells into the circulation [14], [15]. However, little is known about the effects of sevoflurane preconditioning on the MSCs in the ischemic microenvironment.

The first objective of this study was to investigate the effects of sevoflurane preconditioning on MSCs against hypoxia/serum deprivation (H/SD)-induced apoptosis and other physiological characteristics such as migration, proliferation, and expression of HIF-1α, HIF-2α, VEGF, and p-Akt/Akt. HIF1α is one of the major regulators of hypoxic response in most cells and tissues. HIF2α is another regulator closely related to HIF1α. They are usually upregulated together in tumor cells and stem cells under hypoxia environment and play an important role in cellular survival, migration and adhesion [16]–[18]. PI3K/Akt is an important signal transduction pathway, which involves in many cellular physiological activities. Therefore, it is important to investigate the changes of HIF-1α, HIF-2α and PI3K/Akt pathway in the present study.

Transplantation of MSC has long been suggested as a possible logical approach for repair of the damaged nervous system, and neuron-like PC12 cells have been widely studied as a neuronal disease model for in vitro research [19]–[21]. In order to evaluate the effect of sevoflurane preconditioning on the therapeutic potential of MSCs, we next investigate the effects of the co-culturing of sevoflurane preconditioning-MSCs and neuron-like PC12 cells against H/SD-induced apoptosis.

## Materials and Methods

### Culture and Detection of Mesenchymal Stem Cells

Sprague-Dawley rats (weighing about 100±10 g) were obtained from the China Medical University Animal Center. All the procedures were conducted with the approval of the Ethics Committee of China Medical University in accordance with the NIH Guide for the Care and Use of the Laboratory Animals. Rat bone marrow was extracted from the femurs and tibias. The MSCs were cultured in DMEM/F12 supplemented with 10% fetal bovine serum and benzylpenicillin (1×10^5^ U/mL), as described previously [3]. The MSCs were easily isolated in the medium according to their tendency to adhere to plastic. After three days, the flasks were washed twice with phosphate buffered saline (PBS) in order to remove the non-adherent cells. The MSCs were detected by flow cytometric plots and were used for the following experiments at passage 3.

### Hypoxia and Serum Deprivation of Mesenchymal Stem Cells

The in vitro ischemic microenvironment was mimicked for the MSCs by hypoxia and serum deprivation (H/SD) for 24 h. Briefly, MSCs were washed with serum-free medium and were placed in serum-free DMEM/F12. They were then incubated in a sealed, hypoxic GENbox jar fitted with a catalyst (BioMe'rieux, Marcy I'Etoile, France) to scavenge the free oxygen and to maintain the oxygen concentration below 0.1%.

### Sevoflurane Preconditioning of Mesenchymal Stem Cells

MSCs suspension was seeded in sterile 96-well plates at 50 µL per well (10^5^/well) and placed in an airtight chamber (Oxoid anaerobic jar; Oxoid AG, Basel, Switzerland). The Chamber was flushed with an air/5% CO_2_ -mixture for 5 min and then augmented with 2 vol% of sevoflurane for 2 h [22]. Volatile anesthetic concentrations were measured by using the Ohmeda 5330 Agent Monitor (Coast to Coast Medical, Fall River, MA). Control cells were exposed to the air/5% CO_2_ -mixture only.

### Experimental Protocols

Cultured MSCs were divided into three groups. MSCs in the control group were cultured in DMEM/F12 supplemented with 10% fetal bovine serum under normal oxygen supply. MSCs in the H/SD group were cultured without serum and were exposed to hypoxia for 24 h. MSCs in the SP group was preconditioned with sevoflurane. Then, sevoflurane was removed 30 min before H/SD treatment.

### Measurement of Mesenchymal Stem Cells Apoptosis

The MSC apoptosis rate was detected by the fluorescent dye Annexin V-FITC/propidium iodide (PI) apoptosis detection kit (Oncogene, Cambridge, MA) according to the manufacturer's instructions. In brief, cells were rinsed with ice-cold PBS and were resuspended in 200 µL of binding buffer. A total of 10 µL of Annexin V stock solution was added and the cells were incubated at 4°C for 30 min. Finally, 5 µL of PI was added just 5 min before analysis by a FACSC-LSR (Becton-Dickton, USA) equipped with CellQuest software (Becton-Dickton, USA). Ten thousands events were analyzed in each of the samples.

### Measurement of Mitochondrial Membrane Potential

Mitochondrial membrane potential was assessed by the lipophilic cationic probe JC-1 (BioVision, Mountain View, CA), a sensitive fluorescent dye. The red emission of the dye was attributable to a potentially-dependent aggregation in the mitochondria, reflecting mitochondrial membrane potential. Green fluorescence reflected the monomeric form of 5,5′,6,6′-tetrachloro-1,1′,3, 3′-tetraethylbenzimidazolylcarbocyanine iodide-1 (JC-1), appearing in the cytosol after mitochondrial membrane potential depolarization. MSCs were loaded with 10 µmol/L JC-1 for 15 min at 37°C and were immediately analyzed on a FACSC-LSR.

### Analysis of Cell Cycle

For each assay, 1×10^6^ cells were harvested, washed twice with PBS, and fixed in 70% cold ethanol at 4°C overnight. The cells were incubated with 100 µL RNaseA (1 mg/mL) for 30 min at 37°C. A total of 400 µL of the 10 µg/mL PI was added and the solution was kept in the dark for 15 min. Each phase of the cell cycle was measured by a FACSC-LSR and the results were analyzed by using the software ModFit (Verity Software House, Topsham, ME).

### Measurement of Mesenchymal Stem Cells Migration

The MSCs migration was performed in the 24-well transwell migration system with an 8-µm pore size polycarbonate membrane (Corning, Tewksbury, MA). MSCs were trypsinized, washed twice, and transferred to the top chamber with 1% fetal bovine serum DMEM/F_12_ and 0.25% BSA (100 µL, 2×10^4^ cells/well). A total of 500 µL of DMEM/F_12_ containing 10% fetal bovine serum was added to the lower chamber. Following incubation in 21% oxygen or hypoxic condition for 6 h, the cells remaining on the top of the filter were removed and those migrating to the lower surface were fixed in 90% alcohol, followed by a crystal violet staining. The values for migration were obtained by counting three fields per membrane and were represented as the average of six independent experiments conducted over multiple days.

### Western Blot Analysis

After centrifugation, the disposed cells were harvested and rinsed twice with cold PBS. They were then lysed in the ice-cold lysis buffer consisting of 1% Triton X-100, 20 mmol/L HEPES (pH 7.5), 5 mmol/L MgCl_2_, 1 mmol/L EDTA, 1 mmol/L EGTA, 1 mmol/L DTT, 1 mmol/L Phenylmethane sulfonylfluoride, and 1 mg/mL of leupeptin, aprotinin, and pepstatin for 30 min. The centrifugation was performed with heat treatment for 5 min at 100°C. After electrophoresis on 10% SDS-PAGE gel, the proteins were transferred into the polyvinylidene difluoride membranes. The membranes were blocked with 5% skim milk in Tris-buffered saline (TBS) for 1 h at room temperature and were incubated with primary antibodies Akt (sc-55523), phospho-Akt (sc-33437), HIF-1α (sc-53546), and VEGF (sc-1876), purchased from Santa Cruz Biotechnology, CA, HIF-2α (ab20654, Abcam) at 4°C overnight The excessive antibody was washed away by TBS containing 0.1% Tween-20 for 3 times and was replaced by the mouse, rabbit, goat secondary antibody (Santa Cruz Biotechnology, CA) accordingly for 2 h. Semi-quantitation of the scanned films was performed using Quantity One software (Bio-Rad Laboratories, Milan, Italy).

### Culture of Neuron-like PC12 Cells and Co-culture with Mesenchymal Stem Cells

Neuron-like PC12 cells were cultured in DMEM/F12 containing 10% fetal bovine serum with 100 U/ml penicillin-streptomycin. The cells were trypsinized and were transferred into four 6-well plates (2 mL, 2×10^5^ cells/well). The co-culture was performed by transwell co-culture system with a 0.4-µm pore size polycarbonate membrane (Merck Millipore, Billerica, MA) and a total of 1×10^5^ either MSCs or sevoflurane preconditioning-MSCs were seeded in 500 µL of media in the inserts.

Four 6-well plates were randomly divided into four groups, including: control, H/SD (24 h hypoxia and serum deprivation), MSCs (co-culture with non-PC MSCs in the environment of hypoxia and serum deprivation), and SP-MSCs (co-culture with sevoflurane preconditioned MSCs in the environment of hypoxia and serum deprivation) groups. Finally, apoptosis rate was detected by Annexin V-FITC/PI apoptosis detection kit as explained above.

### Statistical Analysis

All data are presented as mean ± SEM. The statistical analysis was performed with Kruskal-Wallis test followed by the Mann-Whitney U test with Bonferroni correction. A *p* value of less than 0.05 was considered statistically significant.

## Results and Discussion

### Detection of Mesenchymal Stem Cells

**Figure 1:**
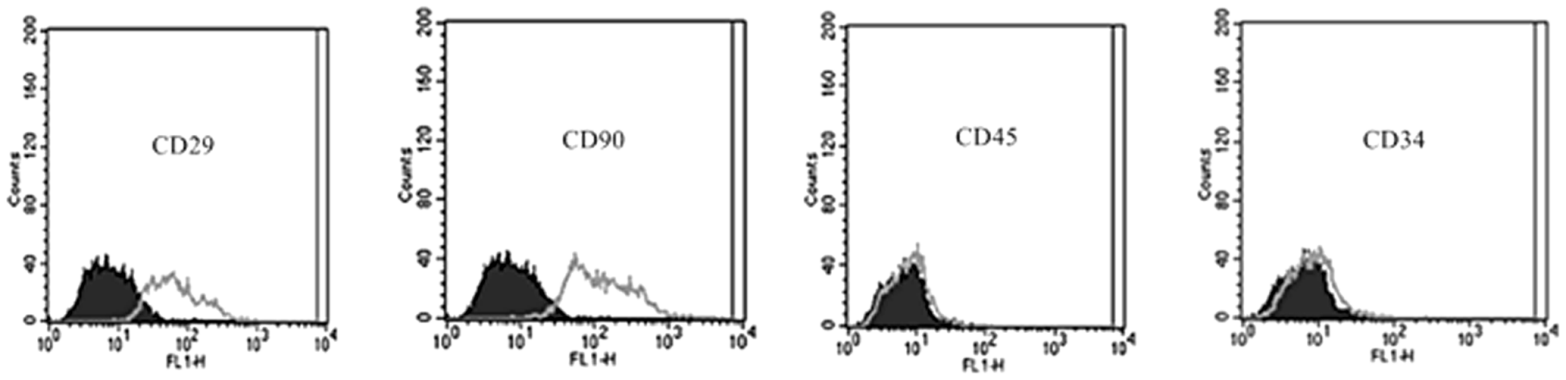
Flow cytometry of MSCs surface markers. X axis represents fluorescence intensity and Y axis expresses cell counts.

The surface markers of MSCs were identified by flow cytometry. The MSCs used in this study positively expressed CD29 and CD90 (90.5% and 94.7%, respectively), and negatively expressed CD34 and CD45 (1.9% and 3.6%, respectively), which were in line with the characteristics of MSCs [23] (Fig. 1).

### Sevoflurane Preconditioning Promoted Survival and Migration of Mesenchymal Stem Cells Against Hypoxia and Serum Deprivation

**Figure 2:**
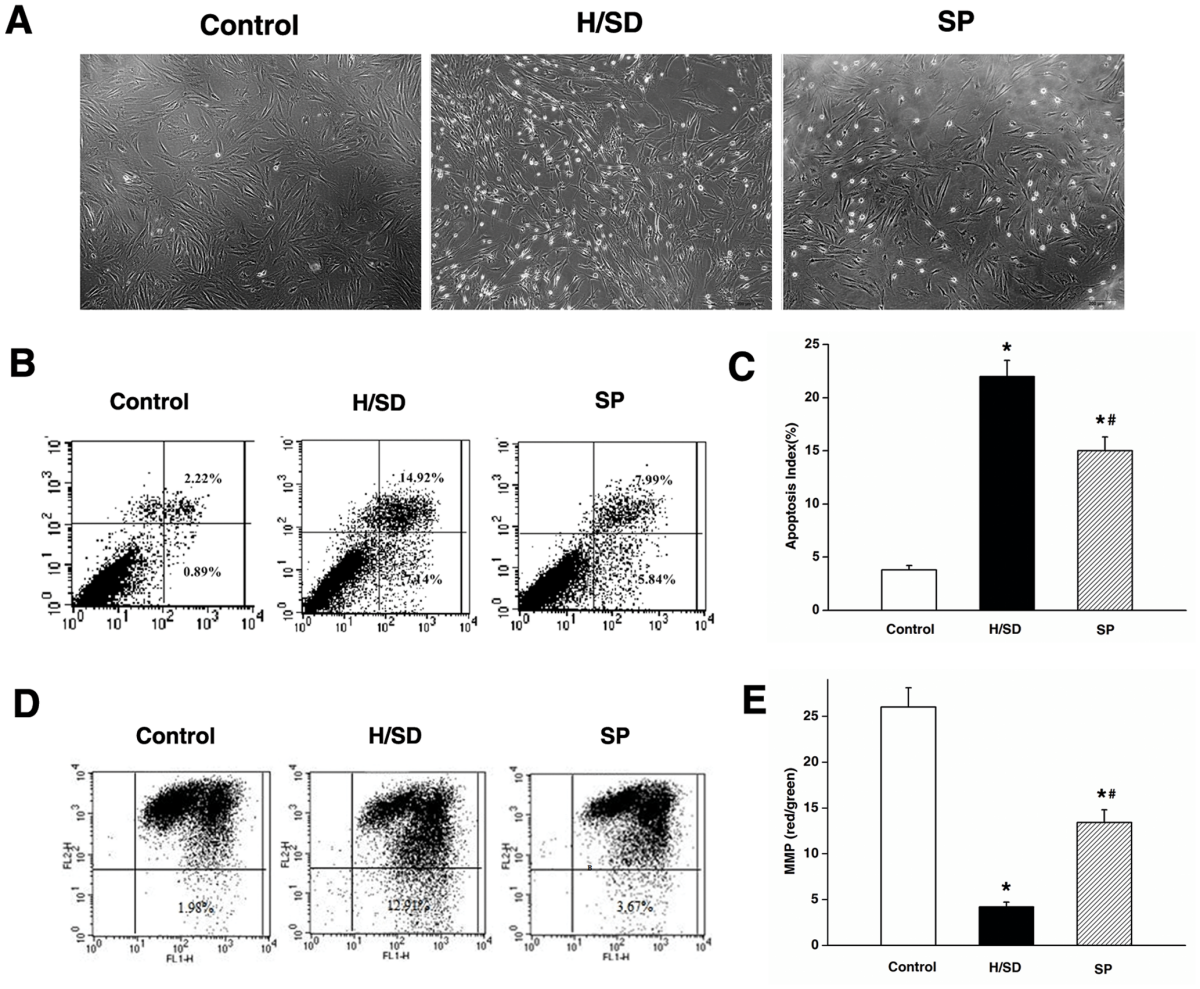
Sevoflurane preconditioning promoted the survival of MSCs against hypoxia and serum deprivation. (A) Representative monolayer morphological images of MSCs under phase-contrast microscopy. In control group, the structure of MSCs was integrated. In H/SD group, the cells emerged as shrinking and rounding. In SP group, these morphological changes were reduced. (B) Flow cytometry of MSCs apoptosis. (C) Quantification of apoptotic cells. (D) Flow cytometry of MSCs mitochondrial membrane potential (MMP). (E) Quantification of MMP. Data are presented as mean ± SEM of six independent experiments. *P<0.05 versus control group. #*P*<0.05 versus H/SD group.

MSCs transplantation has become a promising therapeutic strategy for ischemic diseases [1]–[3], but its therapeutic effect has been hampered because of the death of the transplanted MSCs induced by the ischemic environment [24]. In previous studies, H/SD was chosen as in vitro condition mimicking in vivo ischemic environment to investigate the biological characteristics of MSCs [25]–[28]. In a previous study, H/SD induced programmed death in MSCs [25], which was coincident with our results. Preconditioning with many pharmacological agents might produce a cytoprotection effect on MSCs against hypoxia [6]–[9]. However, there have rarely been researched about the effects of sevoflurane preconditioning on MSCs. In the present study, a 24 h H/SD caused significant morphological changes as well as MSCs apoptosis (*P*<0.05). In contrast, structural integrity was preserved and apoptosis decreased by preconditioning with 2% sevoflurane for 2 h (*P*<0.05) (Fig. 2A–C).

Normal mitochondrial membrane potential is required for mitochondrial function. A variety of cell death signals, such as Ca2+ overload, oxidative stress, and reperfusion injury can open mitochondrial permeability transition pore and cause mitochondrial membrane potential disintegration, breathing strand breakdown, apoptosis-inducing factors (such as cytochrome C) release, and activation of caspase enzymes, follow by apoptosis and cell death. In this study, H/SD induced a loss of mitochondrial membrane potential in the MSCs (*P*<0.05), while sevoflurane preconditioning preserved mitochondrial membrane potential against H/SD (*P*<0.05) (Fig. 2D,E). This observation suggested that sevoflurane preconditioning might reduce MSCs apoptosis via alleviating the mitochondrial membrane potential injury.

**Figure 3:**
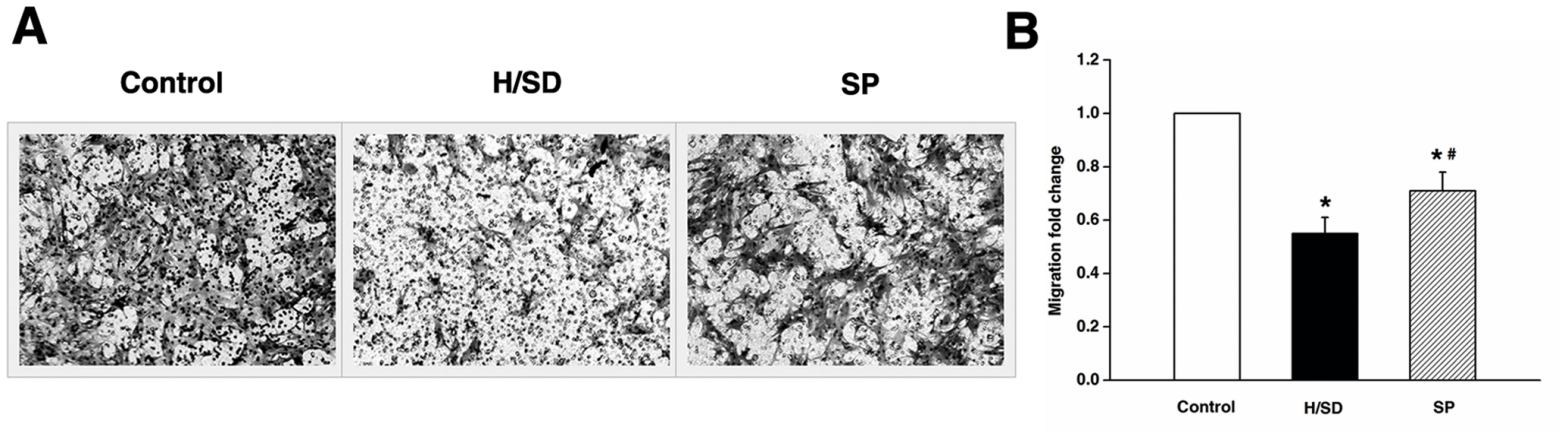
Sevoflurane preconditioning promoted the migration of MSCs against hypoxia and serum deprivation. (A) Representative microscopy images of MSCs penetrating through the transwell chambers (×100 magnification). (B) Quantification of migration. Data are presented as mean ± SEM of six independent experiments. *P<0.05 versus control group. #*P*<0.05 versus H/SD group.

It has been confirmed that hypoxia (1–2%) enhanced the migration capabilities of MSCs [29], [30]. However, the present study found that the number of cells crossing the filter in Transwell test was significantly decreased in H/SD group as compared to the control group (*P*<0.05). The contradictory results might be due to the variations in the oxygen concentration, since severe hypoxia (0.1%) was used in this study. The negative effect was partially reversed by sevoflurane preconditioning in this experiment (*P*<0.05) (Fig. 3A,B).

### Sevoflurane Preconditioning Did Not Promote Proliferation of Mesenchymal Stem Cells Against Hypoxia and Serum Deprivation

**Figure 4:**
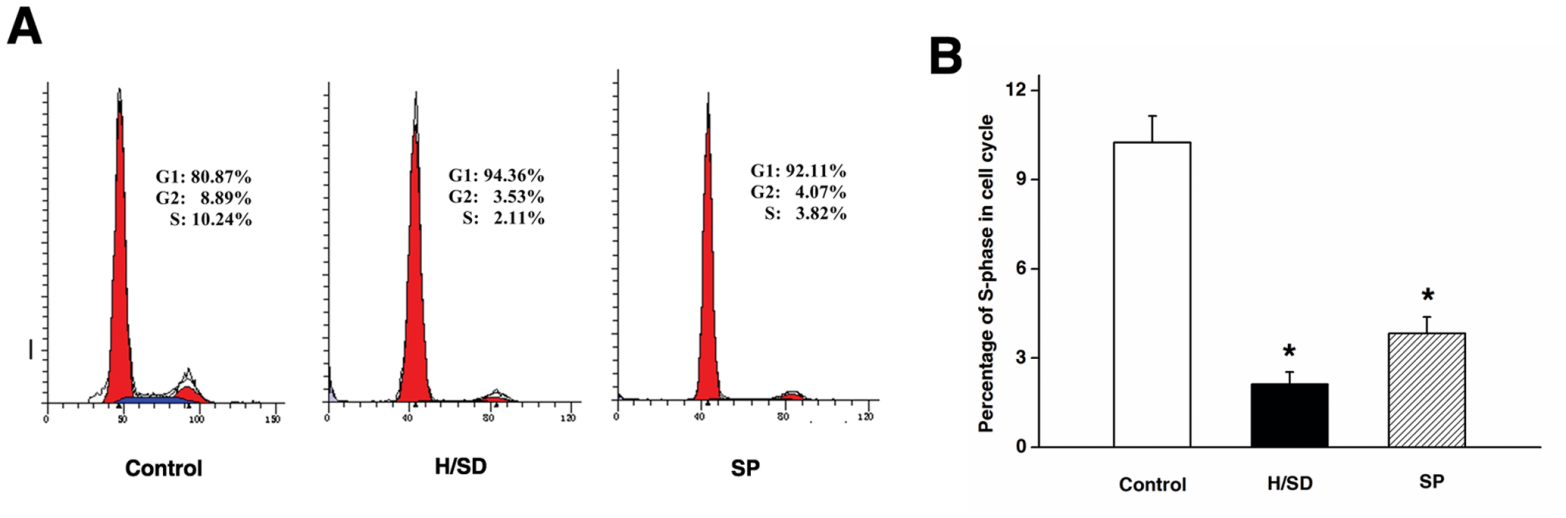
Sevoflurane preconditioning did not promote the proliferation of MSCs against hypoxia and serum deprivation. (A) Flow cytometry of MSCs cell cycle. (B) Quantification of cell cycle. Data are presented as mean ± SEM of six independent experiments. *P<0.05 versus control group.

In this study, a larger percentage of H/SD treated MSCs in the G0/G1 phase was observed. Meanwhile, the percentage of cells in the S phase decreased (*P*<0.05), which showed that H/SD blocked MSCs from entering the DNA synthesis phase and proliferation. Similar results were reported regarding the MSCs slow proliferation and G1 phase accumulation under 1% oxygen as compared with the normal oxygen level [16]. However, there was no significant difference in cell cycle between the H/SD and SP group in the current study (Fig. 4A,B). Therefore, we concluded that sevoflurane preconditioning did not have any obvious effect on MSCs proliferation against H/SD.

### Sevoflurane Preconditioning Increased Expression of HIF-1α, HIF-2α, VEGF, p-Akt/Akt in Mesenchymal Stem Cells Against Hypoxia and Serum Deprivation

**Figure 5:**
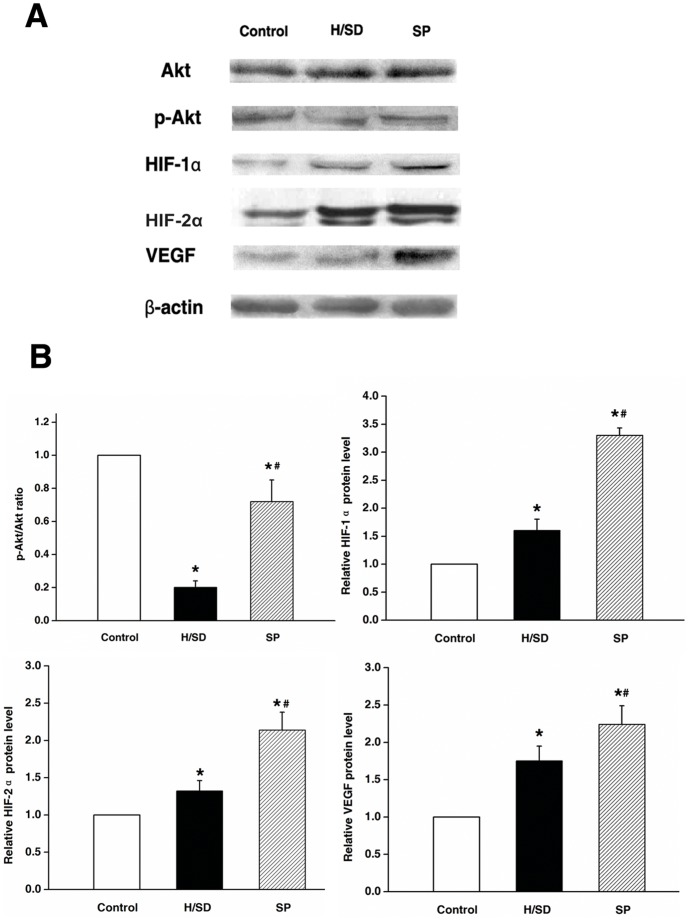
Sevoflurane preconditioning increased the expression of HIF-1α, HIF-2α, VEGF, p-Akt/Akt in MSCs against hypoxia and serum deprivation. (A) Representative western blot of Akt, p-Akt, HIF-1α, HIF-2α and VEGF. (B) Quantitative protein analysis of Akt, p-Akt, HIF-1α, HIF-2α and VEGF. Levels are expressed as ratios to control. Data are presented as mean ± SEM of six independent experiments. *P<0.05 versus control group. #*P*<0.05 versus H/SD group.

HIF-1α and HIF-2α are critical for the cell microenvironment to adapt to low oxygen level through direct or indirect regulation of hundreds of genes, which might enhance survival, migration, and adhesion [16]–[18]. Furthermore, cytoprotection, induced by pharmacological preconditioning through inhalation anesthetics, was partly mediated by regulating HIF-1α nonhypoxic activity [12], [31], [32]. It is necessary to investigate HIF-2α in this condition. We have performed further experiment to check the changes of HIF-2α in our culture, and we found the elevated HIF-2α expression in pharmacological precondition. The results of our study were in agreement with the reports from previous studies showing significant up-regulation of HIF-1α and its downstream gene VEGF in MSCs preconditioned by sevoflurane, meanwhile HIF-2α increased obviously(*P*<0.05) (Fig. 5A,B).

PI3K/Akt is an important part of the signal transduction pathway, which involves migration, adhesion, survival, and many other physiological activities. In a previous study, phosphorylation of Akt was decreased as a result of H/SD induction; however, such induction was reversed by hypoxia preconditioning treatment [33]. In the present study, preconditioning with sevoflurane had a similar effect as that of the hypoxia. Up-regulation of the above cytokines might be responsible for the protective mechanism of sevoflurane preconditioning.

### Sevoflurane Preconditioning Inhibited Apoptosis of Neuron-like PC12 Cells Against Hypoxia and Serum Deprivation

**Figure 6:**
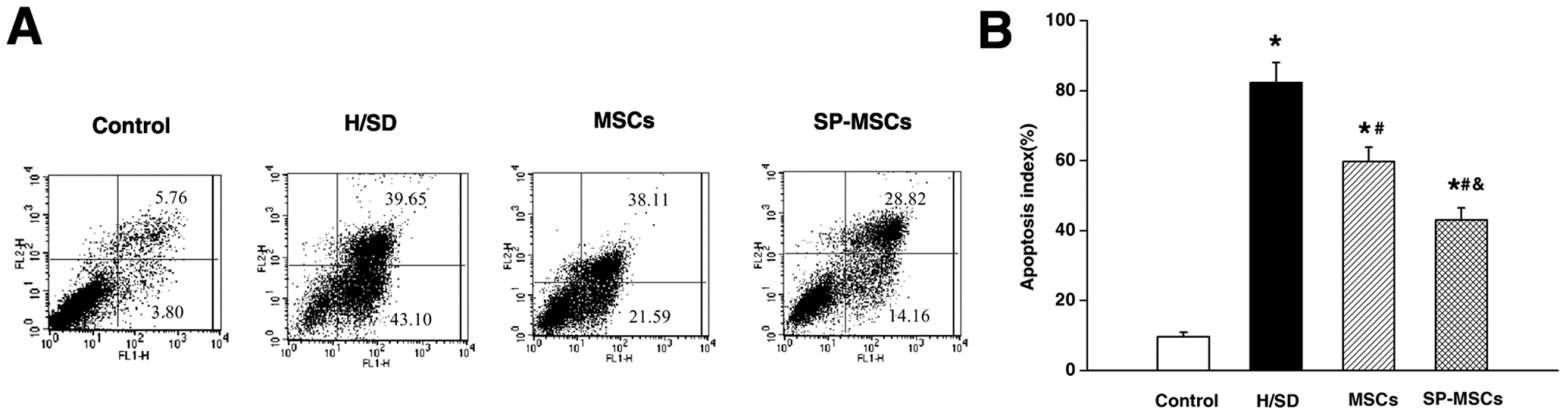
Sevoflurane preconditioning inhibited the apoptosis of neuron-like PC12 cells against hypoxia and serum deprivation. (A) Flow cytometry of neuron-like PC12 cells apoptosis. (B) Quantification of apoptotic cells. Data are presented as mean ± SEM of six independent experiments. *P<0.05 versus control group. #*P*<0.05 versus H/SD group. & *P*<0.05 versus MSCs group.

Recently, several lines of evidence showed that co-culturing with MSCs rescued several kinds of neurocytes from death induced by either oxygen glucose deprivation (OGD) or CoCl_2_ [34]–[37]. Neuron-like PC12 cells have been widely studied as a model for neuronal cells [19]–[21]. In the current study, a 24 h H/SD resulted in an obvious apoptosis in the neuron-like PC12 cells. In contrast, apoptosis decreased by co-culturing with MSCs (*P*<0.05). Moreover, co-culturing with MSCs pretreated with sevoflurane was more effective than non-preconditioned MSCs (*P*<0.05) (Fig. 6A,B). The mechanism for this effect might involve the paracrine factors produced by MSCs, rather than a direct cell contact [38]. Another study confirmed that HIF-1α-AA-modified MSCs protected the PC12 cells from hypoxia-induced apoptosis, partially through VEGF [39]. The current findings showed that sevoflurane preconditioning increased the expression of HIF-1α and VEGF, which might protect the neuron-like PC12 in H/SD microenvironment.

In conclusion, the current study demonstrates that sevoflurane preconditioning improved survival, migration and therapeutic potential of MSCs under ischemic environment. This beneficial effect was mediated in part by upregulation of HIF-1α, HIF-2α, VEGF and p-Akt/Akt.
